# Mediterranean Food Pattern Adherence in a Female-Dominated Sample of Health and Social Sciences University Students: Analysis from a Perspective of Sustainability

**DOI:** 10.3390/nu16223886

**Published:** 2024-11-14

**Authors:** Leandro Oliveira, Ariana Saraiva, Maria João Lima, Edite Teixeira-Lemos, Jwaher Haji Alhaji, Conrado Carrascosa, António Raposo

**Affiliations:** 1 CBIOS (Research Center for Biosciences and Health Technologies), Universidade Lusófona de Humanidades e Tecnologias, Campo Grande 376, 1749-024 Lisboa, Portugal; 2 Research in Veterinary Medicine (I-MVET), Faculty of Veterinary Medicine, Lisbon University Centre, Lusófona University, Campo Grande 376, 1749-024 Lisboa, Portugal; ariana.saraiva@ulusofona.pt; 3 CERNAS Research Centre, Polytechnic University of Viseu, 3504-510 Viseu, Portugal; mjoaolima@esav.ipv.pt (M.J.L.); etlemos3@gmail.com (E.T.-L.); 4 Department of Health Sciences, College of Applied Studies and Community Service, King Saud University, P.O. Box 2455, Riyadh 11451, Saudi Arabia; jalhejjy@ksu.edu.sa; 5 Department of Animal Pathology and Production, Bromatology and Food Technology, Faculty of Veterinary, Universidad de Las Palmas de Gran Canaria, Trasmontaña s/n, 35413 Arucas, Spain; conrado.carrascosa@ulpgc.es

**Keywords:** food sustainability, mediterranean diet, sustainable diets, university students

## Abstract

Background/Objectives: The goal of this pilot study is to evaluate adherence to the Mediterranean Food Pattern (MFP) in a self-selected sample of university students, addressing a perspective of food sustainability. In addition, it seeks to relate adherence to MFP with sociodemographic characteristics and nutritional status. Methods: This is a cross-sectional pilot study whose data collection was carried out by an online questionnaire between January and April 2023. Results: Two hundred and forty-eight students participated—most of them were female (78.2%), had a median of 22 (20; 30) years, resided in the central region of Portugal (42.3%), and were pursuing a degree (73.4%) in a public higher education institution (66.5%). The prevalence of overweight (overweight and obesity) found was 33.1%. Females predominantly used olive oil as their main source of fat (95.9%, *p* = 0.009) and had a higher consumption of sugary drinks (81.4%, *p* = 0.004) compared to males, who reported usage rates of 85.2% and 63.0%, The median score of the Mediterranean Diet Adherence Screener was 7 points, presented with an interquartile range (Q1: 6, Q3: 8), indicating moderate adherence. The analysis showed no differences between the sexes (*p* = 0.087). There was also a negative correlation between adherence to the MFP and the body mass index (*p* = 0.007; r = −0.171). In addition, adherence to the MFP was associated with the area of study and the course attended, with students in health-related fields showing higher adherence. Conclusions: These findings underscore the necessity for targeted interventions aimed at promoting adherence to the MFP among university students, which could contribute to improved health outcomes and enhanced environmental sustainability.

## 1. Introduction

Food sustainability refers to the ability to meet current dietary needs without compromising the ability of future generations to meet theirs. It involves practices that conserve natural resources, reduce waste, and prioritize foods with a lower environmental impact. This is an increasing concern in public health, as it aims to ensure the availability of healthy foods for all while protecting the environment [1]. The processes of food production, processing, distribution, and consumption have implications for both human health and the environment. Moreover, food production inevitably contributes to harmful environmental effects, particularly through factors related to climate, land use, water consumption, and gas emissions. Greenhouse gasses (GHGs) such as carbon dioxide (CO_2_), methane (CH_4_), and nitrous oxide (N_2_O) are known contributors to global warming [2].

In this context, the Mediterranean Food Pattern (MFP) can play a critical role in mitigating climate change effects, as it is recognized as a dietary model that supports health, environmental sustainability, sociocultural values, and economic stability [3]. The MFP is commonly adhered to by populations living in countries bordering the Mediterranean Sea or those influenced by the region. In the 1960s, Ancel Keys first defined the MFP as a low-fat diet rich in vegetable oils, based on the traditional dietary habits of the 20th century in places such as Crete, various regions of Greece, and southern Italy. This diet is characterized by a high consumption of plant-based foods such as cereals, fruits, vegetables, nuts, seeds, and olives. Additionally, it includes a moderate to high consumption of fish and seafood, a moderate intake of eggs, poultry, and dairy products (such as cheese, milk, and yogurt), and a low consumption of red meat. Extra virgin olive oil is the primary source of added fat in this dietary pattern [1,4]. The MFP is continuously evolving, with various adaptations that reflect the diverse food cultures of the Mediterranean region.

According to the National Food and Physical Activity Survey (IAN-AF 2015–2016) [5], there has been a notable deviation from the MFP among the Portuguese population. This shift is reflected in the increased consumption of meat, fish, eggs, dairy products, and cereals, while the intake of vegetables and legumes has declined. Additionally, ultra-processed foods such as cookies, cakes, sweets, salty snacks, pizza, alcoholic beverages, and non-alcoholic beverages (excluding water) now contribute significantly to the diet, accounting for 29% of total consumption [5]. These dietary changes not only deviate from the health-promoting characteristics of the MFP but also pose a challenge to food sustainability. An increased consumption of resource-intensive foods like meat, coupled with a reduced intake of plant-based foods, significantly enlarges the environmental footprint of the Portuguese diet, further straining natural resources and contributing to unsustainable food systems.

A 2020 report from the General Health Directorate [6] revealed that only 26% of the Portuguese population closely adheres to the MFP, with adherence rates even lower among university students [7,8]. The reduced adherence to the MFP correlates with increased dietary practices that demand more natural resources and contribute to environmental degradation. A recent study [8] involving higher education students and researchers in Portugal found that only 8.2% showed high adherence to the MFP. Given the MFP’s recognition for its lower environmental impact compared to other dietary patterns, assessing adherence to the MFP provides insights into the sustainability of food choices among university students. As the MFP is based on the consumption of plant-based foods and limits the intake of resource-intensive foods, a decrease in adherence directly impacts both health outcomes and environmental sustainability.

Entering higher education represents a critical life transition, during which students are exposed to new environments, stress, and often changes in socioeconomic conditions [9]. These factors can lead to the adoption of less healthy and less sustainable eating habits, such as an increased consumption of foods high in sugar, fat, and salt, and a decreased consumption of vegetables [9]. These behaviors further deviate from the MFP and contribute to less sustainable dietary practices.

Therefore, the aim of this pilot study is to evaluate the adherence to the MFP among a sample of university students, with a particular focus on sustainability. By analyzing MFP adherence through the lens of sustainability, the study provides a comprehensive understanding of the relationship between dietary patterns, sociodemographic characteristics, and nutritional status. This pilot study also explores how sociodemographic factors, such as gender, age, and educational background, influence both adherence to the MFP and the broader implications for food sustainability.

Based on the existing literature regarding the relationship between adherence to the MFP, sociodemographic factors, and nutritional status, this study formulates the following hypotheses:

**H1:**  *There are significant differences in the consumption of foods associated with the Mediterranean Food Pattern among university students based on sociodemographic factors*.

**H2:**  *Students enrolled in non-health-related courses exhibit lower adherence to the MFP compared to students in health-related fields*.

**H3:**  *Students with a higher Body Mass Index (BMI) are likely to demonstrate a lower adherence to the MFP*.

**H4:**  *High adherence to the Mediterranean Food Pattern is associated with lower BMI among university students*.

## 2. Materials and Methods

### 2.1. Study Design and Data Collection

This is a cross-sectional pilot study targeting higher education students in Portugal. Data collection took place between January and April 2023, a period chosen to avoid potential seasonal biases in eating habits. This timeframe was selected as it spans across different months, capturing dietary behaviors during both winter and early spring. This ensures a more representative snapshot of students’ food consumption patterns, minimizing the impact of seasonal variations, such as holiday-specific dietary changes or shifts in food availability. An email was distributed to 50 higher education institutions in Portugal, inviting them to share the questionnaire with their students. The distribution request was made through general communication channels, either by sending the survey request to the institution’s main contact email or, when applicable, to the communication office. Only 5 of the 50 institutions contacted agreed to disseminate the questionnaire among their students. Additionally, five institutions responded that they do not disseminate external surveys, while the remaining institutions did not provide any response.

The inclusion criteria for this study were being a higher education student in Portugal and being over 18 years old. Aside from not consenting to participate in the study, no exclusion criteria were established. Data collection was conducted using an online questionnaire through the Google Forms^®^ platform, with all data being self-reported. The questionnaire consisted of four sections: sociodemographic characterization (gender, age, course, and institution attended, course area, anthropometric data), adherence to the MFP, and e-health literacy.

Responses were classified as invalid if participants submitted incomplete questionnaires, particularly if key sections were left unanswered.)

Only the sections related to sociodemographic characterization and adherence to the MFP will be analyzed in this study.

The BMI was calculated using the equation: BMI = weight (kg)/height^2^ (m), utilizing the self-reported weight and height of the participants [10]. The BMI values were then classified according to the World Health Organization’s criteria [11].

Adherence to the Mediterranean Food Pattern was assessed using the Mediterranean Diet Adherence Screener (MEDAS) tool [12], which has been validated for the Portuguese population [13]. This tool comprises 14 questions regarding the consumption or frequency of consumption of foods characteristic of the Mediterranean diet. The final score is obtained by summing the scores of all responses, with a possible range of 0 to 14. Adherence is classified as low (≤5 points), moderate (6 to 9 points), or high (≥10 points) [12,13].

The classification of responses as valid or invalid was based on predefined criteria. A response was deemed invalid if it was incomplete or contained inconsistent information. For example, responses that provided descriptions of foods instead of the requested number of portions were categorized as invalid. The questionnaire was intentionally designed to be straightforward and focused, which helped reduce the likelihood of collecting irrelevant or incomplete data. Additionally, it incorporated a mandatory response mechanism that alerted participants if they attempted to submit the survey without answering all required questions.

While the questionnaire did not include a formal attention-check mechanism, its logical structure and consistency checks facilitated the effective identification and filtering of invalid samples.

### 2.2. Ethical Considerations

This study was conducted in accordance with the ethical standards outlined in the 1964 Declaration of Helsinki and its subsequent comparable ethical guidelines [14]. All relevant information was thoroughly communicated to the study participants. They received informed consent forms detailing the study’s purpose and procedures. Participants were also assured of the confidentiality of their data and informed that the study received approval from the Ethics Commission of the School of Health Sciences and Technologies (P10-22, 7 December 2022).

### 2.3. Statistical Analysis

The statistical analysis was conducted using the Statistical Package for the Social Sciences (SPSS), version 26.0, for Windows. Descriptive statistics were used to provide a comprehensive overview of the participants’ sociodemographic characteristics and nutritional status. These analyses included calculating means, median, and interquartile range (Q1, Q3), and determining the absolute (n) and relative (%) frequencies.

The normality of the distribution of quantitative variables was assessed using the Shapiro–Wilk test, as it is more sensitive to deviations from normality, especially in smaller sample sizes.

Inferential statistical methods were applied to examine the association between variables. Fisher’s exact test or the chi-square test was used to assess the independence between pairs of categorical variables, allowing for the evaluation of associations within the data. The Mann–Whitney U Test or Kruskal–Wallis Test was employed to compare median ranks between independent samples when the variables were continuous and did not follow a normal distribution. Additionally, the Spearman Correlation Coefficient (r) was utilized to evaluate the strength and direction of the linear relationship between pairs of continuous variables.

To evaluate the factors associated with adherence to the MFP, a multinomial logistic regression analysis was conducted. The dependent variable was categorized into three groups: low, moderate, and high adherence to the MFP. The independent variables included the following: sex, area of residence, type of higher education institution, undergoing course, and area of course. The covariables included BMI, and age. The fit of the final model was assessed using the log-likelihood statistic. The overall significance of the model was evaluated through the chi-square test, which compared the log-likelihood of the final model against a null model. Additionally, Pseudo R^2^ values (Cox and Snell, Nagelkerke, and McFadden) were calculated to evaluate the explanatory power of the model. The results were reported as odds ratios (Exp(B)) with 95% confidence intervals to provide insight into the effects of the independent variables on the likelihood of belonging to each adherence category.

The criterion for rejecting the null hypothesis was set at *p* < 0.05. Therefore, when the *p*-value was less than 0.05, the result was considered statistically significant, indicating a meaningful association or difference between the variables under analysis. These methodologies align with those applied in similar recent studies that assessed the same target population, ensuring the robustness and reliability of the findings [15,16].

## 3. Results

Among the five universities that agreed to collaborate, a total of 257 questionnaires were collected. However, nine of these were excluded due to invalid responses, resulting in a final sample comprising 248 participants.

The sociodemographic characteristics of these participants are summarized in Table 1. The majority of participants were female (78.2%), with a median age of 22 years (20; 30). Most participants resided in the Centro region of Portugal (42.3%), were enrolled in undergraduate degree programs (73.4%), and attended public higher education institutions (66.5%). The majority of participants are from health-related courses (39.1%), followed by those from social sciences and humanities (22.6%), exact and life sciences (13.7%), engineering and architecture (8.9%), and the arts (4.8%). Additionally, 10.9% of respondents either did not specify their course area or belong to unspecified fields. The prevalence of overweight (including pre-obesity and obesity) among the sample was 33.1%.

In terms of adherence to the MFP (see Table 2), the majority of participants reported using olive oil as their primary cooking fat (93.5%), with 77.4% consuming fewer than one sugary or carbonated drink per day and 64.5% eating pastry products or sweets less than three times a week. Additionally, 74.2% preferred poultry or rabbit over red meats, and around 90% consumed two or more servings of vegetables, pasta, or rice at least twice weekly.

However, the adherence was lower in other areas: only 16.9% consumed three or more portions of oilseeds weekly, and 23.8% had less than one portion of red or processed meat per day. Moreover, 25.4% reported consuming fewer than four tablespoons of olive oil daily, 31.0% had fewer than three pieces of fruit daily, 39.9% consumed less than three portions of fish or seafood weekly, and 40.3% ate fewer than two portions of vegetables daily. These findings confirmed H1, highlighting significant sociodemographic differences in consumption patterns related to the MFP. Females were more likely to use olive oil as their primary fat source (*p* = 0.009) but also consumed sugary drinks more frequently (*p* = 0.004). Furthermore, students in health-related fields demonstrated better adherence to the MFP compared to their peers in non-health-related courses (*p* = 0.001).

Another factor negatively influencing adherence to the MFP is that 97.6% of participants reported consuming fewer than seven glasses of wine per week. Analysis indicated that females were more likely than males to use olive oil as their primary fat source (*p* = 0.009) and to consume more sugary drinks (*p* = 0.004). The median MEDAS score was 7 (Q1: 6; Q3: 8), reflecting moderate adherence, with no significant differences between genders (see Table 3). The results supported H2, as students enrolled in non-health-related courses demonstrated significantly lower adherence to the MFP compared to those in health-related fields (*p* = 0.001). Additionally, a significant negative correlation was found between adherence to the MFP and BMI (r = −0.171; *p* = 0.007), providing partial support for H3, since students with higher BMI were generally less likely to adhere to the MFP. This correlation further supports H4, as those with higher adherence to the MFP typically had lower BMI values.

Note: Statistical tests considered sociodemographic characteristics (categorical variables) versus the MEDAS (continuous variables). Age and nutritional status (body mass index) were considered continuous variables.

The multinomial logistic regression analysis was conducted to evaluate the factors associated with the adherence to the MFP. The final model demonstrated a log-likelihood of −344.979, yielding a chi-square statistic of 40.983 with a *p* < 0.002, indicating that the model fits the data significantly well. The Pseudo R^2^ values were assessed as follows: Cox and Snell (0.152), Nagelkerke (0.193), and McFadden (0.106), suggesting a moderate explanatory power of the model in describing the adherence to the MFP.

Table 4 presents the coefficients from the multinomial logistic regression, analyzing factors associated with low and moderate adherence to the MFP in comparison to high adherence.

Reference categories: For the adherence to the Mediterranean dietary pattern classes, the reference category is “High”; for area of residence, the reference category is “Lisbon Metropolitan Area/Alentejo/Algarve”; for area of the course, it is “Health”; and for type of higher education institution, it is “Public”.

95% CI: Confidence intervals for Exp(B) indicate the range in which the true effect size may lie.

The analysis revealed significant associations with adherence levels. For low adherence, a positive relationship with BMI was observed (B = 0.145; *p* = 0.065), suggesting that higher BMI may be linked to lower adherence, though this was not statistically significant. Additionally, students enrolled in non-health-related courses showed a significant positive effect (B = 1.858; *p* = 0.007), indicating a higher likelihood of low adherence to the MFP among these students.

In contrast, none of the factors related to moderate adherence, including BMI, age, gender, area of residence, or type of educational institution, demonstrated significant associations, suggesting that these variables did not strongly influence moderate adherence to the MFP. The findings supported H2, as students in non-health-related courses had significantly lower adherence to the MFP compared to their counterparts in health-related fields. The multinomial logistic regression results confirmed this association (B = 1.858; *p* = 0.007). While the data provided partial confirmation for H3, the association between higher BMI and low adherence, although indicative, was not statistically significant (B = 0.145; *p* = 0.065).

## 4. Discussion

This cross-sectional pilot study aimed to evaluate the adherence of university students to the MFP, with a particular focus on food sustainability. Additionally, the study sought to investigate potential associations between adherence to the MFP, sociodemographic characteristics, and the nutritional status of the participants. Overall, the study found a high prevalence of overweight (33.1%) and moderate adherence to the MFP (69.0%), with only 6.5% of students showing high adherence.

These results align with the existing literature, particularly regarding nutritional status. The majority of students in this study had a normal weight (60.5%), which is higher than the figures reported for the general population. In contrast, 34.8% of the Portuguese population is classified as pre-obese, and 22.3% as obese, meaning fewer than 50% of the population is within the normal weight range [5]. However, the prevalence of normal weight among students in this study is lower than that reported in studies conducted with higher education students in Ecuador [17] and Portugal [18], both of which found a prevalence greater than 70%.

These differences may be explained by the dietary habits of higher education students. Several studies, including this one, have reported a negative association between adherence to the MFP and BMI [19,20]. In our analysis, a negative correlation was initially observed, indicating that higher adherence to the MFP was associated with lower BMI values. However, this association dissipated following the application of the multinomial logistic regression model, suggesting that the relationship between adherence to the MFP and BMI may be confounded by other variables not considered in the model, such as physical activity level, total caloric intake, nutritional knowledge, lifestyle habits, and psychological factors. As reported by the Directorate-General for Health in 2020 [6], only 26% of the Portuguese population shows high adherence to the MFP. In this study, the prevalence of high adherence was lower than in several studies involving higher education students in Portugal [7,8,21]. For instance, in Almeida’s study [21], which included 759 students from the University of Porto, 21.3% showed high adherence to the MFP. Similarly, Graça et al. [8] found a prevalence of 8.2% among higher education students and researchers in Portugal. Another study with 305 students from Lusófona University (Lisbon) reported a high adherence rate of 12.5% [7]. On a national scale, a study that included 480 students from higher education institutions and adults from the general population indicated a prevalence of 11% for high adherence to the MFP.

Internationally, a study of 584 Spanish university students reported a prevalence of 36.4% for high adherence to the MFP, with findings differing from this study, as females consumed fewer sugary drinks and more alcoholic beverages than males [22]. In the present study, low wine consumption was recorded, which negatively impacted adherence scores. While this may seem positive given Portugal’s high levels of alcohol consumption [23], it should be interpreted cautiously due to the potential consumption of other alcoholic beverages. A study at a university in Peru reported a high adherence prevalence of 14.2% [19], while a study at a university in Lebanon indicated a 41.0% prevalence of high adherence to the MFP. The Lebanese study also revealed a low consumption of vegetables, fish, and nuts, alongside a preference for white meats and refined products [24], patterns similar to those found in the present study.

Our study provided evidence that gender differences exist in sugary drink consumption among university students in Portugal, with females consuming more sugary drinks than males. This finding is consistent with data from the 2019 National Health Survey conducted by the National Institute of Statistics (Portugal), which also identified variations in sugary drink consumption based on gender, showing that women reported a higher frequency of consumption compared to men among the population aged 15 years and older [25].

In relation to dietary preferences, the living situation of students plays a crucial role in their adherence to the MFP. The transition from living with family to independent living often introduces changes in food choices, with convenience foods becoming more prominent [26,27]. These foods, characterized by their affordability, quick preparation time, and appealing sensory properties, tend to replace traditional, home-cooked meals that align more closely with the MFP. As a result, there is a shift in eating habits towards less healthy, more processed foods, particularly among students living independently. Comparative studies highlight this divergence, showing that students who remain at home maintain healthier eating habits, such as a higher intake of fruits, vegetables, legumes, and fish—staples of the MFP—whereas those who live independently tend to adopt less balanced diets [26,27,28,29].

Another key factor influencing dietary preferences among students is the quality of meals offered in university canteens [29]. There have been several reports criticizing university canteens for providing meals that are low in nutritional quality. Studies have found that these meals often contain excessive amounts of protein, fat, and salt, which are inconsistent with the principles of the MFP. Given that the MFP promotes a diet rich in plant-based foods, lean proteins, and moderation in fat and salt consumption, the disparity in the nutritional content of canteen meals may discourage students from adhering to MFP guidelines. This, in turn, contributes to lower overall adherence to the MFP among university students, as canteens are a primary food source for many students [30].

### 4.1. Adherence to the Mediterranean Food Pattern and Sustainability

The literature indicates that individuals with higher adherence to the MFP are more likely to meet nutritional recommendations. The MFP is plant-based, providing essential nutrients, dietary fiber, and bioactive compounds that contribute to overall well-being, satiety, and the maintenance of a healthy diet [1]. This dietary pattern has demonstrated beneficial effects on lipoprotein levels, endothelial function, insulin resistance, metabolic syndrome, antioxidant capacity, and has shown reductions in myocardial and cardiovascular mortality, as well as cancer incidence, particularly in those who have experienced acute myocardial infarction [1]. Consequently, the MFP has been linked to numerous health benefits, particularly in preventing non-communicable chronic diseases such as cardiovascular disease, obesity, diabetes, and cancer [4].

A study conducted in Tunisia [31], utilizing mathematical diet optimization models, demonstrated that a nutritionally adequate eating pattern that does not consider environmental impact can increase land use and negatively affect biodiversity and soil quality, as well as water consumption. In this context, changes in eating patterns that reduce the intake of animal products—identified as having a greater environmental impact—by replacing them with plant-based foods, can lead to positive environmental effects [32]. Several studies have confirmed that the MFP has a lower environmental impact compared to other dietary patterns [2,4], largely due to its emphasis on plant-based foods and the limited consumption of animal products. This results in reduced land use, lower water consumption, and fewer greenhouse gas emissions compared to other dietary patterns [30,33]. However, given the MFP’s reliance on vegetables, it is essential to prioritize crops with lower water demands to mitigate water resource consumption [32].

From a sociocultural perspective, the MFP is deeply ingrained in the lifestyles of Mediterranean populations and has evolved over centuries, influenced by diverse customs, religious practices, and cultural beliefs, as well as the succession of various dominant civilizations in the region. Frugality, a core principle of the MFP, emphasizes careful food preparation, moderate portion sizes, and the avoidance of waste. These principles are closely tied to the cultural, social, and economic values associated with meals in Mediterranean societies [4]. In these cultures, food transcends mere physiological needs, and communal meals are seen as opportunities for social interaction, pleasure, and enjoyment. They represent daily moments for bonding and shared experiences. Thus, the MFP is not only a reflection of dietary habits but also a manifestation of the rich diversity of Mediterranean food cultures, considered synonymous with the cultural and culinary heritage of the region [3]. Recognizing its significance, UNESCO designated the MFP as an Intangible Cultural Heritage of Humanity in 2010, underscoring its value as a model to be preserved and promoted [4].

Economically, the MFP promotes the preservation and growth of traditional activities by respecting local particularities, thereby fostering a balance between land and society [1]. Given its global visibility, the MFP can play a pivotal role in the sustainable development of small rural Mediterranean areas, particularly by valuing traditional and regional food products [3]. Achieving this objective requires emphasizing local food products and empowering local producers. This necessitates increased transparency and the protection of traditional Mediterranean food products through labeling, adherence to quality standards, and the clear identification of product origins. Furthermore, it is crucial to harmonize tradition, innovation, and sustainability in order to drive the economic and cultural development of Mediterranean regions [3].

From a practical standpoint, adherence to the MFP presents challenges for university students, primarily due to the convenience and accessibility of processed foods, which often come at the expense of environmental sustainability [34]. The shift towards these dietary patterns not only undermines adherence to the MFP but also contributes to increased waste and resource inefficiency. The convenience and accessibility of processed and ready-made foods, combined with a lack of food preparation skills or limited financial resources, often lead to a derivation from the MFP [35]. To improve adherence, interventions targeting university canteens, as well as educational programs promoting the benefits of the MFP and sustainable eating practices, could be beneficial.

This way, while the MFP offers significant health and environmental advantages, its practical implementation among university students faces barriers related to lifestyle changes, economic factors, and institutional food quality [36]. Addressing these barriers requires a multi-faceted approach that promotes both the health and sustainability benefits of the MFP. This includes advocating for public policies that improve food quality in university canteens, implementing educational campaigns to inform students about sustainable eating practices, and addressing economic constraints that limit access to healthier food options. Such interventions are crucial for fostering a culture of sustainability within student populations in Portugal.

### 4.2. Study Limitations and Future Directions

Like all studies, this one has some limitations. It is a cross-sectional pilot study, which does not allow us to determine associations’ temporal direction or generalize the results. Another limitation arises from using a non-probabilistic, non-representative sample of university students, as the participants were self-selected. This self-selection occurs because individuals choose to participate in the study based on their own initiative after receiving the invitation. This may lead to biases, as those who choose to respond may possess different characteristics, motivations, or dietary habits compared to those who do not participate. Therefore, the findings may not accurately reflect the overall population of university students in Portugal.

The gender disproportion in our study may be attributed to the fact that our sample is predominantly composed of students from health and social sciences, fields in which there is a higher representation of females. It is worth noting that in Portugal, approximately 54% of higher education students are female, with even higher rates of feminization in fields such as health and social protection (76.8%), education (76.6%), and social sciences, journalism, and information (65.8%). This suggests that while our sample shows a gender imbalance, it aligns with the trends observed in certain fields of study. Additionally, the sample showed a skewed gender distribution, with 78% of participants identifying as female. This imbalance raises concerns about the representativeness of the sample, as it may not reflect the true gender proportions within the university student population. However, it is worth noting that in Portugal (2023), approximately 60% of higher education students are female [37], indicating that while our sample is skewed, it is not entirely unrepresentative.

Additionally, all data were self-reported rather than independently measured; therefore, there may be discrepancies due to the underestimation or overestimation of variables that could be objectively measured, such as weight and height.

Furthermore, although non-parametric tests such as the Mann–Whitney and Kruskal–Wallis tests are appropriate for analyzing non-normally distributed data, they come with certain limitations. While these tests can effectively identify significant differences between groups, they provide less detailed insights into the underlying distribution of the data compared to parametric tests. As a result, caution is warranted when interpreting *p*-values derived from these analyses, as subtler variations within the data may remain undetected. In this study, the non-parametric analyses were supplemented by a multinomial logistic regression model, enabling a more comprehensive exploration of the relationships among various factors influencing adherence to the Mediterranean Food Pattern (MFP). This multifaceted approach aimed to strengthen the robustness of the findings and highlights the importance of employing diverse analytical methods to achieve a thorough understanding of the dietary habits and associated outcomes of higher education students.

A strength of this study is the use of the MEDAS, a validated tool widely applied in studies across Portugal and the Mediterranean region. This allows for direct comparisons of our results with other population groups within Portugal and internationally.

For future research, several directions should be explored. First, expanding the sample size and diversity is essential to strengthen the robustness of the analyses. A larger dataset would facilitate the examination of more complex relationships, such as interactions between sociodemographic factors and dietary adherence, potentially providing deeper insights into student eating behaviors.

Although we chose not to implement fuzzy set qualitative comparative analysis (fsQCA) in this pilot study, future research with a larger and more diverse sample could benefit from this approach. fsQCA could offer a more systematic understanding of the configurational relationships between factors like lifestyle, socioeconomic background, and health outcomes, contributing to a more comprehensive analysis of MFP adherence. Incorporating fsQCA in future studies may provide a richer, more nuanced understanding of how various factors interact to influence dietary patterns and sustainability practices.

Additionally, future studies should aim to conduct a more representative analysis of university students. It would also be advantageous to explore the relationship between MFP adherence and perceptions of sustainability, incorporating additional variables such as residential area and the distinction between urban and rural environments.

## 5. Conclusions

In this pilot study, the prevalence of overweight exceeding 30% was identified among university students. Approximately 7% of participants demonstrated high adherence to the MFP, while about 25% exhibited low adherence. Students enrolled in health-related courses showed greater adherence to the MFP compared to their peers in other disciplines. Additionally, a negative correlation was initially identified between adherence to the MFP and BMI, suggesting that higher adherence to the MFP was associated with lower BMI values. However, this association diminished after applying the multinomial logistic regression model. This finding indicates that the relationship between MFP adherence and BMI may be influenced by other confounding variables accounted for in the model. Participants primarily used olive oil as their main source of fat and reported consuming fewer sugary drinks daily compared to their counterparts. However, there was also a high consumption of red meat and a low intake of vegetables.

As a pilot study, these findings highlight the importance of developing targeted interventions specifically aimed at university students to promote adherence to the MFP. Such interventions are crucial not only for improving health outcomes associated with this dietary approach but also for encouraging more environmentally sustainable eating habits, with a focus on plant-based foods.

## Figures and Tables

**Table 1:** The sociodemographic characterization of the respondents (*n* = 248).

	*n* (%)		*n* (%)
Sex		Area of the course	
Female	194 (78.2)	Health	97 (39.1)
Male	54 (21.8)	Exact and life sciences	34 (13.7)
Age		Engineering and Architecture	22 (8.9)
Median (P25; P75)	22 (20; 30)	Social Sciences and Humanities	56 (22.6)
Area of residence		Arts	12 (4.8)
North	41 (16.5)	Unknown or unspecified	27 (10.9)
Center	105 (42.3)	Type of Higher Education Institution	
Lisbon Metropolitan Area	62 (25.0)	Public-State	165 (66.5)
Alentejo	9 (3.6)	Public-Non-state	5 (2.0)
Algarve	5 (2.0)	Private	78 (31.5)
Autonomous Region of the Azores	24 (9.7)	Nutritional state ^a^	
Autonomous Region of Madeira	2 (0.8)	Low weight	16 (6.5)
Undergoing course		Normal weight	150 (60.5)
Professional Superior Technical Course	6 (2.4)	Pre-obesity	58 (23.4)
Graduation	182 (73.4)	Obesity	24 (9.7)
Master or Integrated Master	51 (20.6)		
Doctorate	6 (2.4)		
Postgraduate	3 (1.2)		

^a^ Calculated through the body mass index and classified according to the criteria of the World Health Organization [11].

**Table 2:** Adherence to the Mediterranean Food Pattern according to the MEDAS and sex comparison (*n* = 248).

Mediterranean Diet Adherence Screener	Criteria for 1 Point	Total *n* (%)		Female *n* (%)		Male *n* (%)		*p*
		0 Points	1 Point	0 Points	1 Point	0 Points	1 Point	
1. Do you use olive oil as the main cooking fat?	Yes	16 (6.5)	232 (93.5)	8 (4.1)	186 (95.9)	8 (14.8)	46 (85.2)	0.009 *^a^
2. How much olive oil do you consume in one day (including use for frying, seasoning, salads, meals away from home, etc.…)?	≥4 tablespoons	185 (74.6)	63 (25.4)	146 (75.3)	48 (24.7)	39 (72.2)	15 (27.8)	0.650 ^b^
3. How many portions of vegetable products do you consume per day?	≥2	148 (59.7)	100 (40.3)	114 (58.8)	80 (41.2)	34 (63.0)	20 (37.0)	0.578 ^b^
4. How many fruit pieces (including natural fruit juices) do you consume a day?	≥3	171 (69.0)	77 (31.0)	139 (71.6)	55 (28.4)	32 (59.3)	22 (40.7)	0.082 ^b^
5. How many portions of red meat, hamburger or meat products (ham, sausage, etc.…) do you consume a day?	<1	189 (76.2)	59 (23.8)	144 (74.2)	50 (25.8)	45 (83.3)	9 (16.7)	0.165 ^b^
6. How many portions of butter, margarine or cream do you consume per day?	<1	139 (56.0)	109 (44.0)	105 (54.1)	89 (45.9)	34 (63.0)	20 (37.0)	0.247 ^b^
7. How many sugary or carbonated drinks do you consume a day?	<1	56 (22.6)	192 (77.4)	36 (18.6)	158 (81.4)	20 (37.0)	34 (63.0)	0.004 *^b^
8. How many glasses of wine do you drink a week?	≥7 cups	242 (97.6)	6 (2.4)	191 (98.5)	3 (1.5)	51 (94.4)	3 (5.6)	0.119 ^a^
9. How many portions of pulses do you consume per week?	≥3	133 (53.6)	115 (46.4)	103 (53.1)	91 (46.9)	30 (55.6)	24 (44.4)	0.748 ^b^
10. How many portions of fish or seafood do you consume per week?	≥3	149 (60.1)	99 (39.9)	116 (59.8)	78 (40.2)	33 (61.1)	21 (38.9)	0.861 ^b^
11. How many times a week do you consume pastry products or commercial sweets (not homemade), such as cakes, cookies?	<3	88 (35.5)	160 (64.5)	67 (34.5)	127 (65.5)	21 (38.9)	33 (61.1)	0.554 ^b^
12. How many portions of oilseeds (walnuts, almonds, including peanuts) do you consume per week?	≥3	206 (83.1)	42 (16.9)	162 (83.5)	32 (16.5)	44 (81.5)	10 (18.5)	0.726 ^b^
13. Do you prefer to consume chicken, turkey, or rabbit instead of cow, pork, hamburger, or sausage?	Yes	64 (25.8)	184 (74.2)	46 (23.7)	148 (76.3)	18 (33.3)	36 (66.7)	0.153 ^b^
14. How many times a week do you consume vegetables, pasta, rice, or other dishes made with a braised (tomato, onion, leeks or garlic and olive oil)?	≥2	22 (8.9)	228 (91.1)	15 (7.7)	179 (92.3)	7 (13.0)	47 (87.0)	0.277 ^a^
Total Score-Median (P25; P75)	--------	7 (6; 8)		7 (6; 8)		6 (5; 8)		0.087 ^c^

* *p* < 0.05; ^a^ Fisher’s exact test; ^b^ chi-square test; ^c^ Mann–Whitney test.

**Table 3:** Relationship between adherence to the Mediterranean Food Pattern according to the MEDAS and sociodemographic characteristics (*n* = 248).

	Adherence to the Mediterranean Dietary Pattern			*p*
	Low	Moderate	High	
Sex				
Female	42 (21.6)	138 (71.1)	14 (7.2)	0.087 ^a^
Male	19 (35.2)	33 (61.1)	2 (3.7)	
Area of residence				
North	14 (34.1)	24 (58.5)	3 (7.3)	0.337 ^b^
Center	18 (17.1)	79 (75.2)	8 (7.6)	
South	19 (25.0)	53 (69.7)	4 (5.3)	
Islands (Azores and Madeira)	10 (38.5)	15 (57.7)	1 (3.8)	
Undergoing course				
Professional Superior Technical Course or Graduation	51 (27.1)	126 (67.0)	11 (5.9)	0.103 ^a^
Integrated Masters or Postgraduation	10 (16.7)	45 (75.0)	5 (8.3)	
Area of the course				
Health	13 (13.4)	75 (77.3)	9 (9.3)	0.001 *^a^
Non -health	48 (31.8)	96 (63.6)	7 (4.6)	
Type of Higher Education Institution				
Public	44 (26.7)	110 (66.7)	11 (6.7)	0.212 ^a^
Private	17 (20.5)	61 (73.5)	5 (6.0)	
Nutritional state				
Low weight/normal weight	36 (21.7)	118 (71.1)	12 (7.2)	0.007 *^c^ *r*: −0.171
Overweight (pre-obesity and obesity)	25 (30.5)	53 (64.6)	4 (4.9)	
Age	-----------------------------------------------			0.201 ^c^ *r*: 0.081
Total	61 (24.6)	171 (69.0)	16 (6.5)	-----

* *p* < 0.05; ^a^ Mann–Whitney test; ^b^ Kruskal–Wallis test; ^c^ Spearman Correlation.

**Table 4:** Multinomial logistic regression, analyzing factors associated with low and moderate adherence to the MFP in comparison to high adherence.

Adherence to the Mediterranean Dietary Pattern Classes	B	Standard Error	Wald	*df*	*p* *	Exp(B)	95% Confidence Interval for Exp(B)	
							Lower Limit	Upper Limit
Low Adherence to the Mediterranean dietary pattern								
Intercept	−2.055	2.227	0.851	1	0.356			
BMI	0.145	0.079	3.397	1	0.065	1.157	0.991	1.350
Age	−0.063	0.037	2.834	1	0.092	0.939	0.872	1.010
Sex (Male)	0.768	0.874	0.771	1	0.380	2.155	0.388	11.952
Area of residence								
Autonomous Region of the Azores/Madeira	0.113	1.308	0.007	1	0.931	1.120	0.086	14.546
North	0.292	0.998	0.086	1	0.770	1.339	0.189	9.477
Center	−1.010	0.807	1.565	1	0.211	0.364	0.075	1.773
Type of Higher Education Institution								
Private	0.203	0.759	0.071	1	0.789	1.225	0.277	5.428
Area of the course								
Non-Health	1.858	0.694	7.171	1	0.007 *	6.408	1.645	24.957
Undergoing course								
Professional Superior Technical Course/Graduation	0.680	0.769	0.783	1	0.376	1.974	0.437	8.912
Moderate Adherence to the Mediterranean dietary pattern								
Intercept	0.017	2.015	0.000	1	0.993			
BMI	0.079	0.074	1.158	1	0.282	1.083	0.937	1.251
Age	−0.002	0.032	0.002	1	0.962	0.998	0.938	1.062
Sex (Male)	0.203	0.831	0.059	1	0.807	1.225	0.240	6.242
Area of residence								
Autonomous Region of the Azores/Madeira	−0.042	1.251	0.001	1	0.974	0.959	0.083	11.148
North	−0.132	0.932	0.020	1	0.888	0.877	0.141	5.443
Center	−0.280	0.728	0.148	1	0.700	0.755	0.181	3.145
Type of Higher Education Institution								
Private	0.359	0.689	0.271	1	0.603	1.432	0.371	5.524
Area of the course								
Non-Health	0.631	0.617	1.047	1	0.306	1.880	0.561	6.300
Undergoing course								
Professional Superior Technical Course/Graduation	0.359	0.672	0.285	1	0.593	1.432	0.384	5.344

* p < 0.05.

## Data Availability

Data are contained within the article.
